# Risk factors for small-for-gestational-age and preterm births among 19,269 Tanzanian newborns

**DOI:** 10.1186/s12884-016-0900-5

**Published:** 2016-05-17

**Authors:** Alfa Muhihi, Christopher R. Sudfeld, Emily R. Smith, Ramadhani A. Noor, Salum Mshamu, Christina Briegleb, Mohamed Bakari, Honorati Masanja, Wafaie Fawzi, Grace Jean-Yee Chan

**Affiliations:** Ifakara Health Institute, Kiko Avenue, Mikocheni, Dar es Salaam Tanzania; Africa Academy for Public Health, CM Plaza Building, Mwai Kibaki Road, Mikocheni, P.O.Box 79810, Dar es Salaam Tanzania; Department of Global Health and Population, Harvard T. H. Chan School of Public Health, Boston, USA; Department of Nutrition, Harvard T. H. Chan School of Public Health, Boston, USA; Department of Epidemiology, Harvard T. H. Chan School of Public Health, Boston, USA; Department of Medicine, Boston Children’s Hospital, Boston, USA

**Keywords:** Risk factors, Birth weight, Term-SGA, Preterm-AGA, Preterm-SGA, Tanzania

## Abstract

**Background:**

Few studies have differentiated risk factors for term-small for gestational age (SGA), preterm-appropriate for gestational age (AGA), and preterm-SGA, despite evidence of varying risk of child mortality and poor developmental outcomes.

**Methods:**

We analyzed birth outcome data from singleton infants, who were enrolled in a large randomized, double-blind, placebo-controlled trial of neonatal vitamin A supplementation conducted in Tanzania. SGA was defined as birth weight <10th percentile for gestation age and sex using INTERGROWTH standards and preterm birth as delivery at <37 complete weeks of gestation. Risk factors for term-SGA, preterm-AGA, and preterm-SGA were examined independently using log-binomial regression.

**Results:**

Among 19,269 singleton Tanzanian newborns included in this analysis, 68.3 % were term-AGA, 15.8 % term-SGA, 15.5 % preterm-AGA, and 0.3 % preterm-SGA. In multivariate analyses, significant risk factors for term-SGA included maternal age <20 years, starting antenatal care (ANC) in the 3^rd^ trimester, short maternal stature, being firstborn, and male sex (all *p* < 0.05). Independent risk factors for preterm-AGA were maternal age <25 years, short maternal stature, firstborns, and decreased wealth (all *p* < 0.05). In addition, receiving ANC services in the 1^st^ trimester significantly reduced the risk of preterm-AGA (*p* = 0.01). Significant risk factors for preterm-SGA included maternal age >30 years, being firstborn, and short maternal stature which appeared to carry a particularly strong risk (all *p* < 0.05).

**Conclusion:**

Over 30 % of newborns in this large urban and rural cohort of Tanzanian newborns were born preterm and/or SGA. Interventions to promote early attendance to ANC services, reduce unintended young pregnancies, increased maternal height, and reduce poverty may significantly decrease the burden of SGA and preterm birth in sub-Saharan Africa.

**Trial registration:**

Australian New Zealand Clinical Trials Registry (ANZCTR) – ACTRN12610000636055, registered on 3^rd^ August 2010.

## Background

Globally, more than 20 million infants (15.5 % of live births) each year are born low birthweight (LBW) or <2500 g [1], with the vast majority occurring in low and middle income countries (LMICs) [2]. LBW is due to preterm birth (PTB) and intrauterine fetal growth restriction (IUGR) or a combination of both [3, 4]. Small-for-gestational-age (SGA; weight less than 10^th^ percentile for sex and gestational age) is the primary measure for IUGR. It is estimated that of the 135 million babies born in 2010 in LMICs, 21.9 % were term-SGA, 8.1 % were preterm-appropriate for gestational age (AGA) and 2.1 % were preterm-SGA [5].

Preterm and SGA births are both well documented to increase the risk of morbidity and mortality, and newborns who are both preterm and SGA have the highest risk [6, 7]. A multi-country analysis of mortality risk in preterm and SGA births from LMICs determined that, compared to babies born term-AGA, the relative risk for neonatal mortality was 2.44 for term-SGA births, 8.05 for preterm-AGA, and 15.4 for preterm-SGA births [6]. In addition to survival implications, preterm and SGA births have increased risk for malnutrition and life-long complications including impaired neurodevelopment, non-communicable diseases, and psychological or emotional distress [8–10].

Despite a significant body of literature that mortality, morbidity, growth and development outcomes vary for preterm and SGA births, few studies have identified risk factors for combinations of preterm and SGA births [11]. In this analysis we sought to differentiate risk factors for term-SGA, preterm-AGA and preterm-SGA births and to our knowledge this is the first study to do so in Sub-Saharan Africa.

## Methods

### Study design and data collection

This study consist of women and singleton infants enrolled in a randomized double-blind, placebo- controlled neonatal vitamin A supplementation trial conducted in Tanzania between August 2010 and March 2013. Trial recruitment and data collection procedures have been presented elsewhere [12]. Briefly, the trial enrolled participants from urban (Dar es Salaam) and rural (Morogoro) settings in Tanzania. In Dar es Salaam, participants were enrolled at antenatal clinics (ANC) and in labor wards of public health facilities in Kinondoni, Ilala, and Temeke districts. In Morogoro, the study recruited within the Health and Demographic Surveillance System (HDSS) of Ifakara Health Institute which covers approximately 2,400 km^2^ and allowed for enrollment of both health facility and home births.

Newborns were eligible for randomization if they were able to feed orally, were born within the past 72 h, were not previously enrolled in other clinical trials, the family intended to stay in the study area for at least six months post-delivery, and the parents provided written informed consent to participate. A total of 32,843 mothers and their newborns were screened for inclusion in the parent trial. A total of 844 (2.6 %) were excluded for the following reasons: 237 (0.7 %) were not age eligible (>72 h since birth), 38 (0.1 %) were not able to feed orally and 569 (1.7 %) did not plan to reside in the study area for the next six months after delivery. A total of 31,999 newborns were randomized in the trial of which 11,895 resided in Dar es Salaam and 20,104 in Morogoro. There were 30,891 singleton births, and 1,108 were of multiple gestation.

Trained study staff administered a baseline questionnaire to mothers in order to collect information on demographic, socioeconomic, and environmental factors as well as date of mother’s last menstrual period (LMP). We assessed LMP twice, during pregnancy surveillance and at the time of Vitamin A dosing. All infants had their birthweight measured at the time of dosing (at health facility or home) by study staff using calibrated scales with digital screens. Scale calibration with standard weights and weight standardization for all study staff was completed regularly for quality assurance.

### Statistical analysis

We restricted this analysis to 19,269 (62.4 %) singleton infants who had complete data on birth weight and gestational age. Gestational age was calculated from maternal last normal menstrual period (LMP) report and preterm birth was defined as delivery at <37 completed weeks of gestation. SGA was defined as birth weight <10th percentile for gestational age and sex using INTERGROWTH standards [13]. We combined preterm birth and SGA into four mutually exclusive categories; term appropriate-for-gestational age (term-AGA), term small-for-gestational age (term-SGA), preterm appropriate-for-gestational age (preterm-AGA), and preterm small-for-gestational age (preterm-SGA). In sensitivity analyses, we defined preterm as delivery <34 completed weeks of gestation and SGA as <3^rd^ percentile for gestational age and sex using INTERGROWTH standards.

We then examined demographic, socioeconomic, and environmental risk factors of term-SGA, preterm-AGA, and preterm-SGA as compared to reference term-AGA using log-binomial regression models to obtain risk ratio estimates. Variables assessed in univariate and multivariate analyses included location (Dar es Salaam and Morogoro), maternal age (<20, 20–25, 25–30, 30–35 and ≥ 35 years), maternal and paternal education (no formal schooling, some primary, completed primary and secondary plus), wealth quintile, trimester of first ANC visit (1^st^, 2^nd^, 3^rd^ trimester), maternal height (<150, 150.0-154.9, 155.0-159.9, and ≥160.0 cm), parity (first born, 2^nd^–4^th^, and 5^th^ birth or greater), and infant sex. Home versus facility births were only examined in univariate analyses due to issues of causality (preterm births may lead to home births). Wealth index quintile was defined by a principal component analysis of household assets and characteristics (bicycle, radio, mobile phone, television, motorcycle, car, animal ownership, electricity, and roof type) stratified by Dar es Salaam and Morogoro residence. A *priori* we decided to examine potential effect modification of all predictors by location (Dar es Salaam vs. Morogoro). Effect modification was assessed through use of interaction terms with statistical significance determined by the log-rank test. If statistically significant effect modification by site was determined in the univariate model, the interaction term was automatically included in the multivariate model. Missing data were retained using the missing indicator method. All *p*-values were 2–sided with a *p* < 0.05 considered statistically significant. Statistical analyses were performed using SAS v 9.4 (SAS Institute Inc., Cary, NC, USA).

## Results

Baseline characteristics of the 19,269 singleton newborns included in the analysis are presented in Table 1. Briefly, 13,166 newborns (68.3 %) were term-AGA, 3,051 (15.8 %) term-SGA, 2,989 (15.5 %) preterm-AGA, and 63 (0.3 %) were preterm-SGA. Further, 633 newborns (3.3 %) were born <34 weeks gestation and 1,494 newborns (7.8 %) were <3^rd^ percentile for gestational age and sex. The majority of mothers and fathers of newborns in our cohort had at least completed primary school (79.5 and 84.9 % respectively) and most mothers attended their first ANC visit during the second trimester (58.9 %). A total of 1,707 (8.9 %) births took place in the home and there was no difference in mean birthweight for home (mean: 3085 ± 460 g) versus facility births (mean: 3083 ± 476 g) (*p* = 0.87). Baseline characteristics of singleton mothers unable to recall their LMP and who were excluded from the analysis, were similar to singleton mothers who were able to recall their LMP (Appendix 1).

**Table 1:** Baseline characteristics of study participants in total population and stratified by site

	Total Population (*n* = 19,269)
	Mean (SD) or *n* (%)
Residency	
Dar es Salaam region	7,667 (39.8)
Morogoro region	11,602 (60.2)
Maternal age (years)	25.8 ± 5.9
Maternal education	
No formal schooling	1,445 (7.5)
Some primary	1,311 (6.8)
Completed primary	13,294 (69.0)
Secondary and advanced	2,019 (10.5)
Paternal education	
No formal schooling	801 (4.2)
Some primary	926 (4.8)
Completed primary	13,148 (68.2)
Secondary and Advanced	3,209 (16.7)
Trimester of first ANC visit	
1^st^ Trimester	1,858 (9.6)
2^nd^ Trimester	11,339 (58.9)
3^rd^ Trimester	1,630 (23.1)
Maternal height (cm)	155.3 ± 5.2
Infant Sex	
Male	9,963 (51.7)
Female	9,306 (48.3)
Parity	
First born	4,621 (24.0)
2^nd^-4^th^ birth	8,918 (46.3)
5^th^ or greater birth	1,996 (10.4)
Homebirths	1,707 (8.9)
Birth Outcome	
Term-AGA	13,166 (68.3)
Term-SGA	3,051 (15.8)
Preterm-AGA	2,989 (15.5)
Preterm-SGA	63 (0.3)

*AGA* Appropriate for gestational age*, ANC* Antenatal clinic*, SD* Standard deviation*, SGA* Small for gestational age

In Table 2 we presented unadjusted risk factors for term-SGA, preterm-AGA, and preterm-SGA as compared to the reference of term-AGA. Significant risk factors for term-SGA include: younger maternal age, small stature, firstborns, and male sex (*p* < 0.05), with no formal paternal and maternal schooling showing slight protective associations in unadjusted analysis (*p* < 0.05). There was significant interaction between wealth quintile and study site in the crude analysis. Poverty (lowest wealth quintile) was a significant risk factor for term-SGA in Dar es Salaam (RR = 1.36, *p* < 0.001) but was slightly protective in Morogoro (RR = 0.94, *p* = 0.044) (*p*-value for interaction <0.001). Risk factors for preterm-AGA in unadjusted analysis included: younger maternal age, small stature, firstborns, and low maternal and paternal education (*p* < 0.05). We also found that decreased wealth was a significant risk factor for preterm-AGA in both Dar es Salaam and Morogoro (*p*-values 0.001 and <0.001 respectively), but the magnitude of association was significantly greater for Morogoro newborns (*p*-value for interaction: 0.008). In the unadjusted analysis risk factors for preterm-SGA included: both maternal age less than 25 years and older than 30 years as compared to the 25–30 year reference, being firstborn, and decreased maternal height (*p* < 0.05).

**Table 2:** Unadjusted predictors of term-SGA, preterm-AGA, and preterm-SGA as compared to term-AGA reference

Characteristic	Term-AGA	Term-SGA			Preterm-AGA			Preterm-SGA		
	% (*n* = 13,166)	% (*n* = 3,051)	Unadjusted RR (95 % CI)	*p*-value	% (*n* = 2,989)	Unadjusted RR (95 % CI)	*p*-value	% (*n* = 63)	Unadjusted RR (95 % CI)	*p*-value
Maternal age										
< 20 years	12.6	21.1	1.69 (1.54-1.86)	<0.001	16.8	1.37 (1.24–1.52)	<0.001	12.1	2.50 (0.88–7.11)	0.172
20–25 years	30.1	32.3	1.21 (1.11–1.32)	<0.001	33.9	1.20 (1.01–1.31)	<0.001	43.1	3.73 (1.61–8.61)	0.009
25–30 years	28.4	24.2	Ref.		25.5	Ref.		12.1	Ref.	
30–35 years	22.0	16.9	0.91 (0.82–1.02)	0.094	17.7	0.91 (0.82–1.01)	0.070	24.2	3.33 (1.35–8.24)	0.007
≥ 35 years	6.9	5.6	0.96 (0.82–1.12)	0.582	6.1	0.99 (0.85–1.15)	0.920	8.6	4.55 (1.74–11.94)	0.002
Maternal education										
No formal schooling	8.1	6.3	0.78 (0.68–0.90)	<0.001	9.2	1.11 (0.99–1.25)	0.073	9.1	1.22 (0.48–3.09)	0.682
Some primary	7.1	6.9	0.95 (0.83–1.08)	0.448	8.5	1.17 (1.04–1.32)	0.010	7.3	1.12 (0.40–3.14)	0.828
Completed primary	73.2	76.6	Ref.		72.4	Ref.		67.3	Ref.	
Secondary plus	11.6	10.2	0.86 (0.77–0.96)	0.009	10.0	0.89 (0.79–1.00)	0.045	16.4	1.52 (0.74–3.15)	0.255
Paternal education										
No formal schooling	4.4	3.4	0.78 (0.64–0.94)	0.009	5.8	1.23 (1.06–1.41)	0.005	1.8	0.42 (0.06–3.07)	0.394
Some primary	4.9	5.1	1.00 (0.86–1.16)	0.986	6.1	1.16 (1.01–1.34)	0.029	5.5	1.13 (0.35–3.64)	0.840
Completed primary	72.1	74.8	Ref.		73.2	Ref.		70.9	Ref.	
Secondary plus	18.6	16.7	0.89 (0.82–0.98)	0.012	15.0	0.83 (0.75–0.91)	<0.001	21.8	1.19 (0.62–2.28)	0.591
Dar es Salaam wealth quintile										
Q1 (Poorest)	15.9	16.3	1.36 (1.08–1.72)		15.9	1.35 (1.12–1.64)		28.6	3.02 (0.61–14.92)	
Q2	20.7	26.2	1.44 (1.16–1.79)		20.7	1.21 (1.01–1.45)		28.6	2.03 (0.41–10.01)	
Q3	14.9	17.2	1.27 (1.01–1.60)		14.9	1.16 (0.95–1.40)		4.8	0.44 (0.04–4.87)	
Q4	25.6	28.8	1.18 (0.95–1.46)		25.6	1.10 (0.92–1.31)		28.6	1.45 (0.29–7.15)	
Q5 (Richest)	11.1	11.5	Ref.	<0.001*	16.8	Ref.	0.001*	9.5	Ref.	0.464*
Morogoro wealth quintile										
Q1 (Poorest)	16.4	16.7	0.94 (0.83–1.07)		22.2	1.72 (1.49–1.99)		14.7	0.95 (0.30–2.99)	
Q2	21.3	19.2	0.86 (0.76–0.97)		26.7	1.62 (1.41–1.87)		17.7	0.88 (0.30–2.62)	
Q3	17.2	18.4	0.98 (0.87–1.11)		13.8	1.12 (0.95–1.32)		11.8	0.73 (0.21–2.48)	
Q4	20.1	21.7	0.99 (0.88–1.11)		19.2	1.30 (1.12–1.51)		35.2	1.86 (0.73–4.72)	
Q5 (Richest)	21.9	24.0	Ref.	0.044*	15.3	Ref.	<0.001*	20.6	Ref.	0.544*
Trimester of first ANC visit										
1^st^ Trimester	12.9	11.9	0.96 (0.86–1.08)	0.513	11.4	0.90 (0.80–1.01)	0.074	16.0	1.40 (0.65–3.01)	0.393
2^nd^ Trimester	76.5	76.0	Ref.		77.3	Ref.		68.0	Ref.	
3^rd^ Trimester	10.7	12.2	1.09 (0.98–1.22)	0.125	11.3	1.03 (0.92–1.16)	0.567	16.0	1.68 (0.78–3.61)	0.187
Maternal height										
< 150 cm	7.7	12.7	1.90 (1.57–2.31)		9.9	1.48 (1.20–1.83)		23.3	6.36 (1.65–24.46)	
150.0–154.9 cm	33.1	33.5	1.31 (1.11–1.54)		36.8	1.33 (1.13–1.55)		36.7	2.35 (0.66–8.41)	
155.0–159.9 cm	37.9	38.2	1.31 (1.11–1.53)		36.5	1.18 (1.01–1.39)		30.0	1.69 (0.46–6.21)	
≥ 160.0 cm	21.3	15.6	Ref.	<0.001*	16.8	Ref.	<0.001*	10.0	Ref.	0.011
Parity										
First born	26.2	42.5	1.71 (1.59–1.84)	<0.001	31.9	1.25 (1.16–1.34)	<0.001	49.1	2.53 (1.51–4.22)	<0.001
2^nd^–4th birth	59.8	49.0	Ref.		55.9	Ref.		43.4	Ref.	
5^th^ or greater	14.0	8.6	0.78 (0.68–0.89)	<0.001	12.2	0.95 (0.85–1.06)	0.386	7.6	0.73 (0.26–2.06)	0.553
Infant sex										
Male	51.0	54.0	1.10 (1.03–1.17)	0.004	52.4	1.05 (0.98–1.12)	0.168	50.8	0.99 (0.61–1.62)	0.971
Female	49.0	46.0	Ref.		47.6	Ref.		49.2	Ref.	
Place of birth										
Home	8.5	7.4	0.89 (0.79–1.01)	0.06	11.8	1.34 (1.22–1.48)	<0.001	12.7	1.56 (0.75–3.27)	0.236
Facility	91.5	92.6	Ref.		88.2	Ref.		87.3	Ref.	

*p-value for trend

*AGA* Appropriate for gestational age, *ANC* Antenatal clinic, *CI* Confidence interval, *SGA* Small for gestational age, *RR* Relative risk

**Fig. 1:**
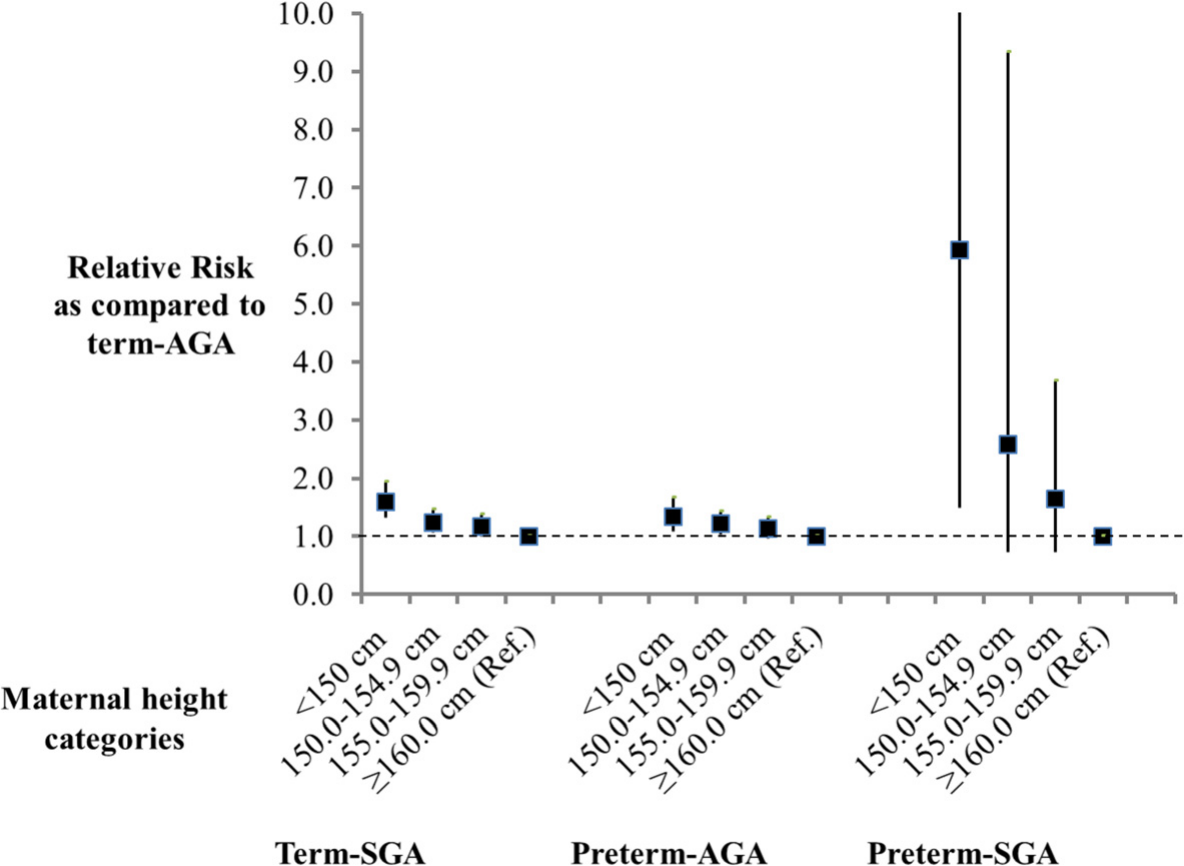
Relative risk of term-SGA, preterm-AGA and preterm-SGA for maternal height categories as compared to ≥160.0 cm reference

In the multivariate analysis, we identified several important risk factors for term-SGA, preterm-AGA, and preterm-SGA as compared to the term-AGA reference (Table 3). Significant, independent risk factors for term-SGA include: maternal age <20 years (*p* = 0.002), late ANC first visit in 3^rd^ trimester as compared to 2^nd^ trimester (*p* = 0.025), decreased maternal stature under 160 cm (*p* < 0.001), being firstborn (*p* < 0.001), and male sex (*p* = 0.007). Significant protective factors for term-SGA included maternal secondary education (*p* = 0.018) and no formal paternal schooling (*p* = 0.028). For preterm-AGA, significant risk factors included: maternal age <25 years, decreased maternal stature (*p* < 0.001), and being firstborn (*p* = 0.003). In addition, attending ANC for the first time in the first trimester as compared to second trimester (*p* = 0.009) and paternal secondary education were associated with significantly reduced risk of preterm-AGA. Decreased wealth was a significant risk factor for preterm-AGA in Morogoro (*p* < 0.001) and the results indicated a similar, but smaller in magnitude and not statistically significant trend in Dar es Salaam (*p* = 0.076) (*p*-value for interaction: 0.024). For preterm-SGA, significant independent risk factors included maternal age >30 years, firstborns, and decreased maternal height (*p* = 0.042). Figure 1 illustrates the magnitude of risk of term-SGA, preterm-AGA, and preterm-SGA for maternal height. Women with short stature have an increased risk of all three adverse pregnancy outcomes.

**Table 3:** Multivariate adjusted predictors of term-SGA, preterm-AGA, and preterm-SGA as compared to term-AGA reference

Characteristic	Term-SGA		Preterm-AGA		Preterm-SGA	
	Adjusted RR (95 % CI)	*p*-value	Adjusted RR (95 % CI)	*p*-value	Adjusted RR (95 % CI)	*p*-value
Maternal age						
< 20 years	1.19 (1.06–1.32)	0.002	1.24 (1.10–1.39)	<0.001	0.90 (0.29–2.80)	0.860
20–25 years	1.07 (0.98–1.17)	0.135	1.16 (1.06–1.27)	0.001	2.22 (0.93–5.29)	0.072
25–30 years	Ref.		Ref.		Ref.	
30–35 years	0.98 (0.89–1.09)	0.769	0.93 (0.83–1.03)	0.150	3.33 (1.33–8.35)	0.010
≥ 35 years	1.09 (0.93–1.28)	0.292	1.00 (0.86–1.18)	0.954	4.66 (1.39–15.67)	0.013
Maternal education						
No formal schooling	0.88 (0.76–1.02)	0.091	1.03 (0.91–1.17)	0.651	1.64 (0.61–4.41)	0.327
Some primary	1.01 (0.88–1.15)	0.913	1.11 (0.98–1.26)	0.108	1.38 (0.47–4.05)	0.559
Completed primary	Ref.		Ref.		Ref.	
Secondary plus	0.87 (0.77–0.98)	0.018	0.95 (0.84–1.07)	0.387	1.33 (0.58–3.01)	0.499
Paternal education						
No formal schooling	0.80 (0.66–0.98)	0.028	1.08 (0.93–1.26)	0.319	0.39 (0.05–3.06)	0.374
Some primary	0.98 (0.84–1.14)	0.799	1.07 (0.93–1.24)	0.334	1.15 (0.34–3.87)	0.826
Completed primary	Ref.		Ref.		Ref.	
Secondary plus	0.98 (0.89–1.08)	0.689	0.85 (0.77–0.95)	0.004	1.21 (0.57–2.55)	0.625
Dar es Salaam wealth quintile						
Q1 (Poorest)	1.26 (1.00–1.60)		1.21 (1.00–1.46)		3.00 (0.59–15.27)	
Q2	1.34 (1.08–1.67)		1.10 (0.92–1.32)		2.06 (0.41–10.39)	
Q3	1.18 (0.94–1.49)		1.08 (0.89–1.31)		0.43 (0.04–4.73)	
Q4	1.15 (0.93–1.42)		1.08 (0.90–1.29)		1.40 (0.28–6.97)	
Q5 (Richest)	Ref.	0.057*	Ref.	0.076*	Ref.	0.827*
Morogoro wealth quintile						
Q1 (Poorest)	1.00 (0.90–1.12)		1.49 (1.32–1.68)		1.67 (0.67–4.25)	
Q2	0.95 (0.86–1.06)		1.39 (1.24–1.56)		1.34 (0.54–3.29)	
Q3	1.00 (0.90–1.11)		1.13 (1.00–1.28)		0.66 (0.22–1.99)	
Q4	0.95 (0.86–1.05)		1.23 (1.09–1.37)		1.64 (0.73–3.67)	
Q5 (Richest)	Ref.	0.432*	Ref.	<0.001*	Ref.	0.842*
Trimester of first ANC visit						
1^st^ Trimester	0.98 (0.88–1.09)	0.712	0.86 (0.76–0.96)	0.009	1.33 (0.61–2.89)	0.475
2^nd^ Trimester	Ref.		Ref.		Ref.	
3^rd^ Trimester	1.13 (1.02–1.26)	0.025	1.06 (0.94–1.19)	0.323	1.83 (0.84–9.98)	0.127
Maternal height						
< 150 cm	1.60 (1.33–1.94)		1.34 (1.09–1.66)		5.92 (1.50–23.30)	
150.0–154.9 cm	1.24 (1.06–1.46)		1.22 (1.04–1.43)		2.58 (0.72–9.32)	
155.0–159.9 cm	1.17 (1.00–1.37)		1.13 (0.97–1.33)		1.64 (0.44–6.08)	
≥ 160.0 cm	Ref.	<0.001*	Ref.	<0.001*	Ref.	0.042
Parity						
First born	1.56 (1.42–1.70)	<0.001	1.15 (1.05–1.26)	0.003	3.21 (1.63–6.33)	0.001
2^nd^–4th birth	Ref.		Ref.		Ref.	
5^th^ or greater	0.75 (0.64–0.86)	<0.001	0.99 (0.87–1.12)	0.869	0.45 (0.14–1.42)	0.174
Infant sex						
Male	1.09 (1.02–1.16)	0.007	1.05 (0.98–1.12)	0.134	1.02 (0.62–1.67)	0.947
Female	Ref.		Ref.		Ref.	

*p-value for trend

*AGA* Appropriate for gestational age, *ANC* Antenatal clinic*, CI* Confidence interval, *SGA* Small for gestational age*, RR* Relative risk

Sensitivity analyses utilizing a preterm definition of <34 weeks and SGA as defined by <3^rd^ percentile are presented in Appendix 2: Table 5 and Appendix 3: Table 6. We were unable to present risk factors for preterm-SGA in sensitivity analyses utilizing these more extreme definitions due to the small number of infants in this category (*n* = 2). Young maternal age, late ANC, short maternal stature, starting ANC in 3^rd^ trimester, and firstborns remained significant risk factors for term-SGA (<3^rd^ percentile) and were of similar magnitude (Appendix 2: Table 5). In addition, maternal secondary education was significantly associated with reduced risk of term-SGA (<3^rd^ percentile). As for preterm (<34 weeks) –SGA, young maternal age and decreased wealth in Morogoro region remained significant predictors (Appendix 3: Table 6). In addition, decreased wealth in Dar es Salaam approached statistical significance as a predictor of preterm (<34 weeks) –SGA.

## Discussion

In this analysis we found both common and distinct risk factors for term-SGA, preterm-AGA, and preterm-SGA births. Short maternal stature and being firstborn were significant risk factors for all three pregnancy outcomes. Young maternal age was a risk factor for both term-SGA and preterm-AGA, while advanced maternal age over 30 years was associated with increased risk for preterm-SGA. Additional risk factors for term-SGA were starting ANC late in the third trimester of pregnancy and male sex. Poor socioeconomic status for women residing in the rural setting increased the risk for preterm-AGA, while starting ANC early in the first trimester of pregnancy was protective.

We determined that young maternal age was associated with an increased risk of term-SGA and preterm-AGA, whereas maternal age >30 years was associated with increased risk of preterm-SGA. A similar pattern was also seen in a study differentiating risks of preterm and SGA births in Nepal, but results were not statistically significant [14]. The leading biological mechanisms to explain the high risk of adverse birth outcomes of young mothers include maternal-fetal competition for nutrients or incomplete physical maturation which might contribute to adverse neonatal outcomes [15]. As for the relationship of maternal age above 30 years, older women have increased risk for congenital abnormalities and pregnancy comorbidities including hypertension and gestational diabetes which can increase the risk of preterm and SGA [16, 17]. Family planning interventions to prevent unintended early pregnancies may reduce the risk of preterm-AGA and term-SGA births and their consequences, while access to essential newborn care is critical for pregnant women of advanced maternal age due to risk of preterm-SGA births, which carry the highest risk of mortality.

Consistent with other studies which examined the association of maternal anthropometry with pregnancy outcomes [18, 19], we found that short maternal stature, an indicator of chronic malnutrition, was independently associated with increased risk for term-SGA, preterm-AGA and preterm-SGA. The association of short maternal stature and adverse pregnancy outcomes is likely to be due to a combination of increased risk of cephalo-pelvic disproportion and an indicator of poor supply of nutrients to the fetus due to maternal malnutrition [20, 21]. Our results also confirm the association between short maternal stature and pregnancy outcomes appears to be stronger for SGA as compared to PTB [22].

Being firstborn was associated with risk of all combinations of preterm and SGA birth outcomes. This finding matches with findings from rural Nepal and a meta-analysis examining parity and maternal age as risk factors for PTB and SGA [11, 14]. From the meta-analysis, it was suggested that the association with PTB was largely driven by young maternal age and/or its interaction with null parity. Starting ANC late in the third trimester of pregnancy was associated with increased risk for term-SGA as compared to the second trimester, while starting ANC early in the first trimester of pregnancy reduced risk of preterm-AGA. The mechanism leading to this association may be a combination of early detection and management of pregnancy related health conditions and increased duration of standard pregnancy interventions like iron and folic acid supplementation and sulphadoxine pyrimethamine (SP) for prevention of malaria in pregnancy [23].

There are few limitations to our analysis. First, exclusion of newborns that were unable to feed orally in the parent trial may have underestimated the burden of PTB and SGA, as well as biased associations determined in this paper. Nevertheless, only 38 infants were excluded from the trial due to inability to feed orally, which is likely to have a negligible effect on our estimates based on 19,269 births. In addition, we were limited by data collected in the primary neonatal vitamin A supplementation trial and did not have information to evaluate or control for other known risk factors for adverse birth outcomes including: pre-pregnancy BMI, weight gain during pregnancy, history of chronic diseases like hypertension and diabetes, birth intervals, and previous history of PTB and SGA [24, 25]. Lastly, preterm and SGA were defined using maternal report of LMP, which likely lead to some misclassification. Nevertheless, errors in maternal report of LMP are likely not systematically related to both birth outcomes and risk factors of interest which would lead to underestimation of the associations of interest.

## Conclusion

This study identified common and unique risk factors for term-SGA, preterm-AGA and preterm-SGA ranging from anthropometric, economic, demographic and behavioral factors. Some of the risk factors like late ANC attendance, young maternal age at conception, short maternal stature, and poverty are potentially modifiable, and provide an opportunity to improve birth outcomes. In addition, due to high burden of preterm and SGA births in both urban and rural settings in Tanzania, it is vital to advocate for universal access to essential newborn care within the country and similar settings. Overall, targeted combinations of prevention and treatment interventions during pregnancy may decrease the burden of preterm and SGA births and provide substantial reductions in child mortality, morbidity, growth and developmental delay in resource-limited settings.

### Ethics approval and consent to participate

The study protocol was approved by the Institutional Review Boards of the Harvard T.H. Chan School of Public Health, Ifakara Health Institute, Medical Research Coordinating Council of Tanzania, and by the WHO Ethical Review Committee. Individual informed consent was sought from at least one parent of every infant who was enrolled in the trial.

### Consent for publication

Not applicable.

### Availability of data and materials

Please contact Professor Wafaie Fawzi (mina@hsph.harvard.edu) or Dr. Honorati Masanja (hmasanja@ihi.or.tz) for data requests.

## Appendix 1

**Table 4:** Comparison of baseline characteristics of singleton trial participants who were able to recall LMP versus those who were not able to recall LMP

	Able to Recall LMP (*n* = 19,269)	Unable to recall LMP (*n* = 11,622)
Maternal age (years)	25.8 ± 5.9	26.0 ± 5.9
Maternal education		
No formal schooling	1,445 (7.5)	1,111 (9.6)
Some primary	1,311 (6.8)	920 (7.9)
Completed primary	13,294 (69.0)	8,054 (69.3)
Secondary and advanced	2,019 (10.5)	882 (7.6)
Paternal education		
No formal schooling	801 (4.2)	607 (5.2)
Some primary	926 (4.8)	787 (6.8)
Completed primary	13,148 (68.2)	8,149 (70.1)
Secondary and Advanced	3,209 (16.7)	1,446 (12.4)
Wealth quintile		
Q1 (Poorest)	3,073 (16.0)	2,451 (21.1)
Q2	4,085 (21.2)	2,582 (22.2)
Q3	3,124 (16.2)	1,807 (15.6)
Q4	4,440 (23.0)	2,301 (19.8)
Q5 (Richest)	3,432 (17.8)	1,631 (14.0)
Trimester of first ANC visit		
1^st^ Trimester	1,858 (9.6)	817 (7.0)
2^nd^ Trimester	11,339 (58.9)	7,730 (66.5)
3^rd^ Trimester	1,630 (23.1)	1,186 (10.2)
Maternal height (cm)	155.3 ± 5.2	155.7 ± 4.5
Infant Sex		
Female	9,306 (48.3)	5,390 (46.4)
Male	9,963 (51.7)	6,232 (53.6)
Parity		
First born	4,621 (24.0)	2,953 (25.4)
2^nd^-4^th^ birth	8,918 (46.3)	5,287 (45.4)
5^th^ or greater birth	1,996 (10.4)	1,618 (13.9)

*ANC* Antenatal clinic, *LMP* Last Menstrual period

## Appendix 2

**Table 5:** Unadjusted and multivariate adjusted predictors of term (≥37 weeks) -SGA (<3^rd^ percentile)

	Term-AGA (>3 %)	Term (≥37 weeks) -SGA (<3^rd^ percentile)				
Characteristic	% (*n* = 14,738)	% (*n* = 1,479)	Unadjusted RR (95 % CI)	*p*-value	Multivariate adjusted RR (95 % CI)	*p*-value
Maternal age						
< 20 years	13.4	21.7	1.78 (1.54–2.05)	<0.001	1.21 (1.02–1.43)	0.027
20–25 years	30.3	32.9	1.25 (1.09–1.43)	<0.001	1.07 (0.94–1.23)	0.304
25–30 years	28.0	23.8	Ref.		Ref.	
30–35 years	21.5	17.1	0.94 (0.80–1.10)	0.428	1.00 (0.85–1.17)	0.979
≥ 35 years	6.8	4.4	0.78 (0.60–1.00)	0.054	0.86 (0.65–1.13)	0.270
Maternal education						
No formal schooling	8.0	5.5	0.68 (0.54–0.85)	0.001	0.78 (0.62–0.99)	0.042
Some primary	7.0	7.1	0.97 (0.80–1.18)	0.787	1.09 (0.89–1.33)	0.406
Completed primary	73.5	77.1	Ref.		Ref.	
Secondary plus	11.5	10.3	0.87 (0.74–1.03)	0.100	0.84 (0.70–1.00)	0.054
Paternal education						
No formal schooling	4.2	3.7	0.85 (0.65–1.11)	0.235	0.94 (0.71–1.25)	0.679
Some primary	5.0	4.8	0.93 (0.73–1.18)	0.541	0.95 (0.75–1.21)	0.668
Completed primary	75.8	72.3	Ref.		Ref.	
Secondary plus	15.7	18.5	0.82 (0.72–0.95)	0.007	0.83 (0.71–0.97)	0.016
Dar es Salaam wealth quintile						
Q1 (Poorest)	15.0	16.3	1.16 (0.82–1.64)		1.02 (0.71–1.45)	
Q2	22.3	29.1	1.36 (1.00–1.86)		1.24 (0.90–1.70)	
Q3	17.2	14.5	0.91 (0.64–1.31)		0.85 (0.59–1.22)	
Q4	31.0	26.6	0.92 (0.67–1.27)		0.91 (0.67–1.26)	
Q5 (Richest)	14.5	13.5	Ref.	0.020*	Ref.	0.717*
Morogoro wealth quintile						
Q1 (Poorest)	16.9	17.0	0.96 (0.79–1.15)		1.22 (1.00–1.49)	
Q2	21.8	18.2	0.81 (0.67–0.97)		0.94 (0.77–1.13)	
Q3	17.8	19.3	1.03 (0.86–1.23)		1.19 (1.00–1.44)	
Q4	20.9	21.5	0.98 (0.82–1.16)		1.08 (0.90–1.29)	
Q5 (Richest)	22.7	24.0	Ref.	0.153*	Ref.	0.324*
Trimester of first ANC visit						
1^st^ Trimester	12.9	11.6	0.91 (0.77–1.09)	0.300	0.89 (0.74–1.05)	0.169
2^nd^ Trimester	76.4	76.0	Ref.		Ref.	
3^rd^ Trimester	10.8	12.5	1.15 (0.97–1.36)	0.100	1.23 (1.00–1.55)	0.014
Maternal height						
< 150 cm	8.1	13.5	1.94 (1.47–2.57)		1.65 (1.25–2.19)	
150.0–154.9 cm	33.4	31.2	1.17 (0.92–1.48)		1.09 (0.86–1.38)	
155.0–159.9 cm	37.8	39.0	1.18 (1.02–1.61)		1.18 (0.94–1.49)	
≥ 160.0 cm	20.7	16.3	Ref.	0.001*	Ref.	0.005*
Parity						
First born	27.6	45.1	1.87 (1.69–2.07)	<0.001	1.79 (1.57–2.05)	<0.001
2^nd^–4th birth	58.9	46.2	Ref.		Ref.	
5^th^ or greater	13.4	8.7	0.81 (0.67–0.99)	0.039	0.88 (0.71–1.10)	0.267
Infant sex						
Male	52.1	46.4	0.81 (0.75–0.90)	<0.001	0.82 (0.74–0.90)	<0.001
Female	47.9	53.6	Ref.		Ref.	

**p*-value for trend

*AGA* Appropriate for gestational age*, ANC* Antenatal clinic*, CI* Confidence interval*, SGA* Small for gestational age*, RR* Relative risk

## Appendix 3

**Table 6:** Unadjusted and multivariate adjusted predictors of preterm (<34 weeks)-SGA (<10^th^ percentile)

	Term-AGA (>10 %)	Preterm (<34 weeks)-SGA (<10th percentile)				
Characteristic	% (*n* = 15,522)	% (*n* = 633)	Unadjusted RR (95 % CI)	*p*-value	Multivariate adjusted RR (95 % CI)	*p*-value
Maternal age						
< 20 years	13.1	19.1	1.66 (1.31–2.10)	<0.001	1.46 (1.11–1.92)	0.007
20–25 years	30.6	35.4	1.33 (1.08–1.64)	0.006	1.27 (1.02–1.57)	0.029
25–30 years	28.0	24.1	Ref.		Ref.	
30–35 years	21.5	15.3	0.84 (0.65–1.08)	0.174	1.00 (0.85–1.17)	0.979
≥ 35 years	6.8	6.1	1.05 (0.74–1.50)	0.787	0.86 (0.65–1.13)	0.270
Maternal education						
No formal schooling	8.3	8.1	0.99 (0.74–1.33)	0.942	0.92 (0.67–1.26)	0.599
Some primary	7.3	6.9	0.96 (0.70–1.32)	0.789	0.86 (0.62–1.20)	0.382
Completed primary	73.1	72.0	Ref.		Ref.	
Secondary plus	13.0	11.3	1.16 (0.91–1.47)	0.233	1.24 (0.95–1.61)	0.114
Paternal education						
No formal schooling	4.6	4.9	1.06 (0.73–1.54)	0.774	0.96 (0.64–1.43)	0.838
Some primary	5.1	6.5	1.26 (0.91–1.76)	0.161	1.15 (0.82–1.61)	0.430
Completed primary	72.3	72.0	Ref.		Ref.	
Secondary plus	18.0	16.7	0.94 (0.75–1.17)	0.565	0.94 (0.74–1.20)	0.626
Dar es Salaam wealth quintile						
Q1 (Poorest)	15.1	21.9	1.76 (1.16–2.68)		1.73 (1.11–2.69)	
Q2	22.5	20.1	1.11 (0.73–1.71)		1.07 (0.69–1.68)	
Q3	17.0	16.4	1.20 (0.77–1.88)		1.15 (0.73–1.82)	
Q4	30.7	29.9	1.21 (0.81–1.81)		1.20 (0.79–1.80)	
Q5 (Richest)	14.7	11.7	Ref.	0.028*	Ref.	0.053*
Morogoro wealth quintile						
Q1 (Poorest)	17.1	27.1	2.22 (1.56–3.16)		1.82 (1.23–2.68)	
Q2	22.3	21.8	1.40 (0.97–2.02)		1.20 (0.81–1.78)	
Q3	16.7	13.6	1.17 (0.77–1.76)		1.05 (0.69–1.62)	
Q4	19.9	19.6	1.40 (0.96–2.04)		1.24 (0.84–1.84)	
Q5 (Richest)	21.0	14.5	Ref.	<0.001*	Ref.	0.006*
Trimester of first ANC visit						
1^st^ Trimester	12.7	10.7	0.86 (0.65–1.14)	0.293	0.81 (0.61–1.08)	0.153
2^nd^ Trimester	76.6	76.2	Ref.		Ref.	
3^rd^ Trimester	10.7	13.0	1.21 (0.93–1.57)	0.152	1.24 (0.96–1.61)	0.803
Maternal height						
< 150 cm	7.9	12.9	1.57 (1.01–2.42)		1.42 (0.92–2.20)	
150.0–154.9 cm	33.8	34.6	1.01 (0.71–1.42)		0.95 (0.67–1.34)	
155.0–159.9 cm	37.9	31.7	0.83 (0.58–1.17)		0.80 (0.56–1.13)	
≥ 160.0 cm	20.5	20.8	Ref.	0.099*	Ref.	0.613*
Parity						
First born	26.9	34.6	1.36 (1.15–1.62)	<0.001	1.12 (0.90–1.39)	0.318
2^nd^–4th birth	59.2	55.1	Ref.		Ref.	
5^th^ or greater	13.8	10.3	0.80 (0.61–1.07)	0.130	0.85 (0.62–1.17)	0.333
Infant sex						
Male	51.3	52.1	1.03 (0.89–1.21)	0.663	1.04 (0.90–1.22)	0.572
Female	48.8	47.9	Ref.		Ref.	

**p-*value for trend

*AGA* Appropriate for gestational age*, ANC* Antenatal clinic, *CI* Confidence interval, *SGA* Small for gestational age, *RR* Relative risk

## Abbreviations

AGA: appropriate-for-gestational age
ANC: antenatal care clinic
HDSS: Health and demographic surveillance system
IUGR: intrauterine fetal growth restriction
LBW: low birthweight
LMICs: low and middle income countries
LMP: last normal menstrual period
PTB: preterm birth
RR: relative risk
SGA: small-for-gestational age
WHO: World health organization
